# Effectors of the spindle assembly checkpoint are confined within the nucleus of Saccharomyces cerevisiae

**DOI:** 10.1242/bio.037424

**Published:** 2019-06-10

**Authors:** Lydia R. Heasley, Jennifer G. DeLuca, Steven M. Markus

**Affiliations:** 1 Department of Environmental and Radiological Health Sciences, Colorado State University, Fort Collins, Colorado 80523, USA; 2 Department of Biochemistry and Molecular Biology, Colorado State University, Fort Collins, Colorado 80523, USA

**Keywords:** Spindle assembly checkpoint, Budding yeast, Mitotic checkpoint complex, Mitosis, Mitotic exit network

## Abstract

The spindle assembly checkpoint (SAC) prevents erroneous chromosome segregation by delaying mitotic progression when chromosomes are incorrectly attached to the mitotic spindle. This delay is mediated by mitotic checkpoint complexes (MCCs), which assemble at unattached kinetochores and repress the activity of the anaphase promoting complex/cyclosome (APC/C). The cellular localizations of MCCs are likely critical for proper SAC function, yet remain poorly defined. We recently demonstrated that in mammalian cells, in which the nuclear envelope disassembles during mitosis, MCCs diffuse throughout the spindle region and cytoplasm. Here, we employed an approach using binucleate yeast zygotes to examine the localization dynamics of SAC effectors required for MCC assembly and function in budding yeast, in which the nuclear envelope remains intact throughout mitosis*.* Our findings indicate that in yeast, MCCs are confined to the nuclear compartment and excluded from the cytoplasm during mitosis.

## Abstract (precis)

**Summary:** The effectors of the spindle assembly checkpoint are confined with the nuclear compartment of budding yeast, and cannot exchange between nuclei in a binucleate zygote.

## INTRODUCTION

Accurate chromosome segregation during mitosis is facilitated by the spindle assembly checkpoint (SAC), a conserved signaling pathway that monitors the attachment of chromosomes to the mitotic spindle via kinetochores, large protein complexes that assemble upon centromeric DNA (Musacchio, 2015; Musacchio and Salmon, 2007; Kops and Shah, 2012). Kinetochores form load-bearing attachments to spindle microtubules to facilitate: (1) chromosome alignment during prometaphase, and (2) segregation of sister chromatids during anaphase (Kops and Shah, 2012). The SAC monitors kinetochore-microtubule attachment status and delays anaphase onset in the presence of unattached kinetochores, thus ensuring that when anaphase occurs, all chromosomes are positioned to be equally inherited into the two daughter cells (Musacchio, 2015; Hoyt et al., 1991). Through the activity of kinetochore-associated SAC effectors (e.g. Mad1, Mad2, Mad3, Bub1 and Bub3), unattached kinetochores generate a ‘wait anaphase’ signal, comprised of mitotic checkpoint complexes (MCCs; Sudakin et al., 2001; Yamaguchi et al., 2016; Faesen et al., 2017). MCCs inhibit the activity of the anaphase promoting complex/cyclosome (APC/C), an E3 ubiquitin ligase, by sequestering the activating subunit Cdc20 (Yamaguchi et al., 2016; Faesen et al., 2017). By inhibiting APC/C activity, MCCs prevent the degradation of key mitotic substrates such as Cyclin B and Securin, and thus delay anaphase onset (Musacchio, 2015). In addition to Cdc20, the MCC is composed of Mad2, Mad3 (the homolog of human BubR1) and Bub3 (Sudakin et al., 2001; Yamaguchi et al., 2016; Faesen et al., 2017). Mad1 and Bub1 catalyze the assembly of MCCs at unattached kinetochores, and are required for SAC function (Ji et al., 2017; Faesen et al., 2017).

Even a single unattached kinetochore is sufficient to delay anaphase onset (Rieder et al., 1995; Collin et al., 2013). Upon attachment of all kinetochores to spindle microtubules, MCC assembly ceases and cells rapidly enter anaphase (Rieder et al., 1995; Collin et al., 2013; Dick and Gerlich, 2013). The mechanisms that enable the SAC to maintain a robust mitotic delay and yet also enable its rapid silencing remain unclear. One hypothesis explaining the robust nature of the checkpoint postulates that a single unattached kinetochore can catalyze the formation of sufficient levels of MCCs to maintain an arrest (Ciliberto and Shah, 2009). Cellular MCC concentrations are dictated by the rates of MCC assembly and disassembly, and the cellular volume that MCCs occupy during mitosis (Ciliberto and Shah, 2009). Alteration of these parameters perturb the strength of a SAC arrest (Ciliberto and Shah, 2009; Kyogoku and Kitajima, 2017; Galli and Morgan, 2016). For example, several recent studies have demonstrated that the high frequency of chromosome segregation errors observed in cells with large cytoplasmic volumes (e.g. embryonic cells and oocytes) results from the dilution of MCCs (Kyogoku and Kitajima, 2017; Galli and Morgan, 2016). Our recent work characterized the mobility of MCCs within mammalian cells, and helped to define the relationship between cell volume and SAC activity (Heasley et al., 2017). Using fused mammalian cells (with two mitotic spindles; Heasley et al., 2017), we demonstrated that both spindles in a fused cell entered anaphase synchronously, suggesting that MCCs can in fact move throughout the cytoplasm and between spindles. The parameters of MCC mobility in mammalian cells are dictated, in part, by the fact that these cells perform ‘open’ mitosis (Arnone et al., 2013; Boettcher and Barral, 2013). This raises the question of how the presence of a nuclear envelope might impact the mobility of these effectors and thus checkpoint function. Specifically, we wondered how the presence of the nuclear envelope might alter the localization patterns and dynamics of SAC effectors throughout mitosis. We chose to use the budding yeast *Saccharomyces cerevisiae* to investigate these questions as these cells perform closed mitosis, and their SAC effectors are highly conserved with those found in metazoans. Here, we demonstrate that in contrast to mammalian cells, MCCs in yeast remain confined within the nucleus during mitosis.

## RESULTS AND DISCUSSION

### Mad1 and Bub1 are retained in the nucleus throughout the cell cycle

**Fig. 1.**
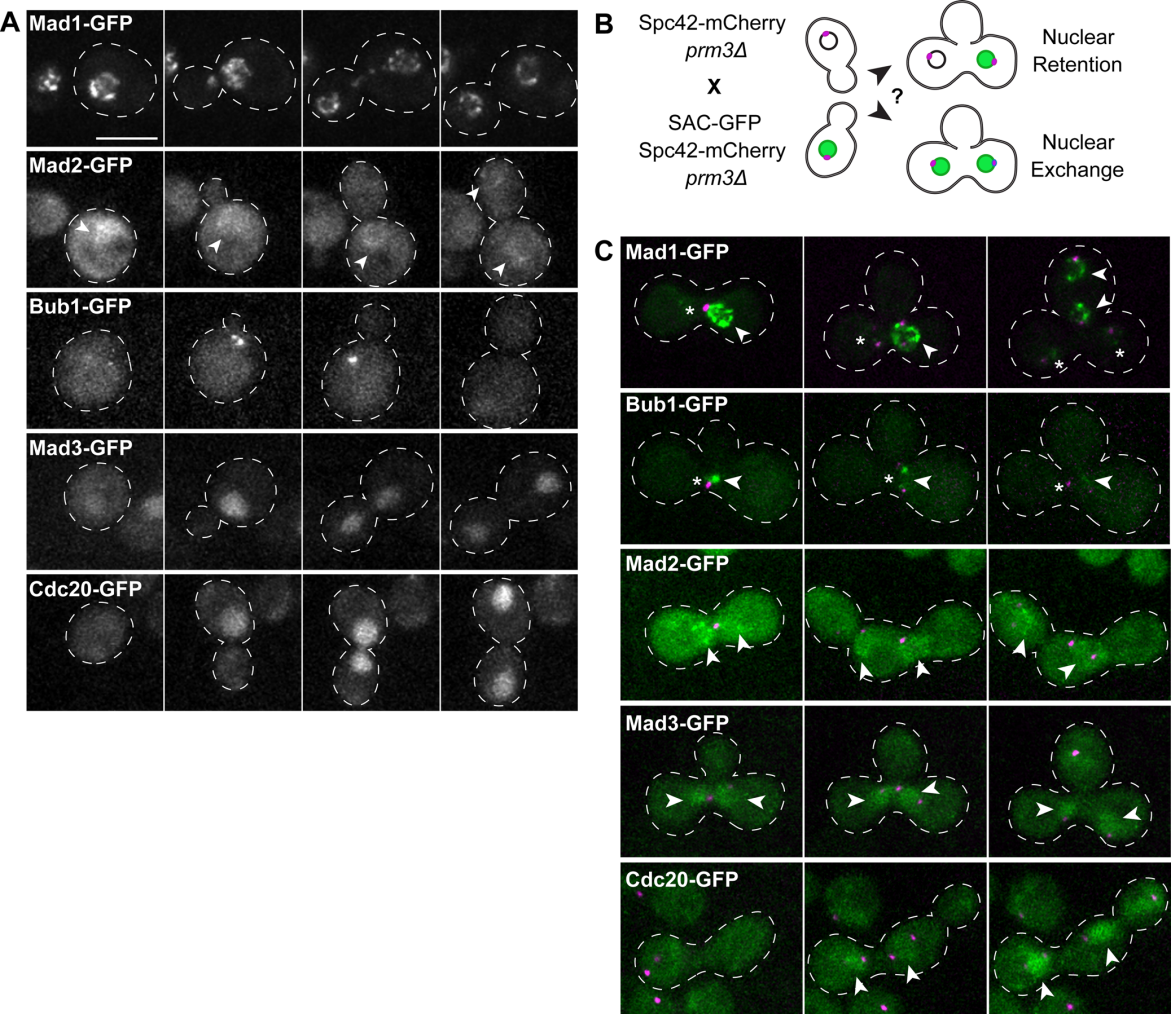
**SAC effectors exhibit variable localization dynamics throughout the cell cycle.** (A) Representative time-lapse images of haploid cells expressing Mad1-, Mad2-, Bub1-, Mad3- or Cdc20-GFP as they progress through mitosis. Arrowheads in Mad2-GFP panel denote nuclear envelope localization. (B) Schematic depicting experimental approach to determine the localization dynamics of test SAC effectors in binucleate zygotes. (C) Representative time-lapse images of binucleate zygotes expressing Spc42-mCherry (magenta) and indicated test SAC-GFP (green). Images were acquired every 5 min. Scale bar: 5 μm for all. Note that Mad1- and Bub1-GFP are only apparent in their respective SAC-GFP-expressing nuclei (arrowheads in Mad1 and Bub1 panels delineate Mad1- and Bub1-GFP-containing nuclei prior to and following nuclear division), and lacking in the others (asterisks). Fluorescence due to Mad2-, Mad3-, and Cdc20-GFP is apparent in both nuclei shortly after cell fusion (see arrowheads in each respective panel).

We first sought to determine the localization of key SAC effectors during closed mitosis in budding yeast. Specifically, we chose to assess the localization of the following molecules throughout the cell cycle in haploid yeast cells: Mad1, Mad2, Mad3, Bub1 and Cdc20 (Fig. 1A; also see Materials and Methods). In agreement with previous studies (Cairo et al., 2013; Iouk et al., 2002; Scott et al., 2005; Rodriguez-Bravo et al., 2014), we found that Mad1- and Mad2-GFP localized to the nuclear envelope throughout the cell cycle, although the latter also exhibited diffuse localization in both the cytoplasm and nucleoplasm. Bub1-GFP localized as one or two foci per cell transiently during early mitosis, and was undetectable throughout the rest of the cell cycle. Previous studies have demonstrated that these Bub1-GFP foci likely coincide with kinetochores (Gillett et al., 2004). Finally, Mad3- and Cdc20-GFP exhibited diffuse cytoplasmic and nuclear localization that became enriched in the nucleus as cells progressed into mitosis.

These results indicate that different SAC effectors localize to distinct cellular landmarks (i.e. the nuclear envelope, kinetochores, or simply diffuse within the nucleoplasm) at different times throughout the cell cycle. However, these observations did not reveal what impact the nuclear envelope – which persists throughout mitosis in budding yeast – has on the ability of these molecules, and complexes assembled from them (e.g. the MCC) to exchange between the nucleus and the cytoplasm. To address this directly, we employed an approach using binucleate yeast cells. The premise for this approach is as follows: if a nuclear-localized factor diffuses from the nucleoplasm to the cytoplasm, we will observe its localization in both nuclei of a binucleate cell. If, on the other hand, a nuclear-localized factor is retained in its respective nucleus, we will only observe it in that nucleus, but not the neighboring nucleus with which it shares a common cytoplasm (see Fig. 1B). We generated binucleate yeast cells by mating haploid strains each deleted for *PRM3* (*prm3*Δ), which is required for nuclear fusion during mating (Shen et al., 2009). The resulting zygotes contained two nuclei in a shared cytoplasm (Fig. 1B). To determine if a specific SAC effector exchanged between nucleoplasm and cytoplasm, we mated *prm3*Δ cells expressing a GFP-tagged SAC effector to *prm3*Δ cells that did not express the GFP fusion (Fig. 1B,C). To delineate the approximate nuclear position, these cells also expressed Spc42-mCherry, which marks the nuclear envelope-embedded spindle pole bodies (SPBs; see Materials and Methods). We imaged cells from the time of fusion until anaphase onset, and assessed to what extent, if any, the GFP-SAC effector localized to both nuclei. Interestingly, we found that both Mad1- and Bub1-GFP remained exclusively enriched in just one of the two nuclei (presumably the SAC-GFP-expressing nucleus), and were never observed in the both nuclei (Fig. 1C; nucleus lacking SAC-GFP fluorescence marked with an asterisk; *n*≥10 cells). This suggests that these proteins – which are both catalysts of MCC assembly – remain confined within the nucleus throughout the cell cycle, and are not shuttled into the cytoplasm. In contrast, both nuclei in binucleate zygotes accumulated the MCC complex components Mad2-, Mad3- and Cdc20-GFP prior to spindle assembly (as assessed by SPB separation; see arrowheads in Fig. 1C; *n*≥10 cells), suggesting that these factors are indiscriminately imported into both nuclei. These findings indicate that checkpoint effectors in yeast exhibit distinct localizations throughout the cell cycle, and also exhibit different nuclear import/export properties.

### Cdc20 is retained in the nucleus subsequent to import

Our observations indicate that Mad1 and Bub1 – neither of which are subunits of the MCC (Sudakin et al., 2001; Yamaguchi et al., 2016; Faesen et al., 2017; Izawa and Pines, 2015) – are nuclear confined; on the other hand, Mad2, Mad3 and Cdc20 – all of which are subunits of the MCC – appear to be indiscriminately imported into both nuclei. However, it was unclear from these results whether intact MCCs are also free to exchange between nuclei subsequent to their catalytic assembly, which takes place at unattached kinetochores within the nucleus. To determine if this is the case, we used a strategy that would permit us to quantitatively determine the degree of nuclear-cytoplasmic exchange of Cdc20 subsequent to its import. We chose Cdc20 as a marker because a subset of the cellular Cdc20 pool is integrated into the MCC. To this end, we employed combined FLIP (fluorescence loss in photobleaching)-FRAP (fluorescence recovery after photobleaching) analysis of Cdc20-GFP-expressing binucleate cells. If Cdc20-GFP diffuses between nuclear and cytoplasmic compartments, then a photobleached nucleus will recover fluorescence intensity over time due to the import of Cdc20-GFP from the cytoplasm (FRAP). At the same time, the unbleached nucleus will lose fluorescence intensity due to: (a) the export of Cdc20-GFP from this nucleus, and (b) the import of photobleached (i.e. non-fluorescent) Cdc20-GFP molecules from the cytoplasm that originated from the photobleached nucleus (FLIP).

As proof-of-concept, we performed FLIP-FRAP with Arx1, a known nuclear shuttling factor that is similar in molecular weight to Cdc20 (Belaya et al., 2006) and thus likely exhibits similar passive nuclear import/export parameters. Studies in yeast have demonstrated that proteins smaller than 50 kDa can passively diffuse through the nuclear pore, while proteins larger than 50 kDa rely on karyopherin-mediated import and export (Shulga et al., 2000; Shulga and Goldfarb, 2003). Both Arx1 and Cdc20 are greater than 50 kDa (65 and 67 kDa respectively; 94 kDa and 96 kDa with GFP), and thus likely require active transport to transit through nuclear pores.

**Fig. 2.**
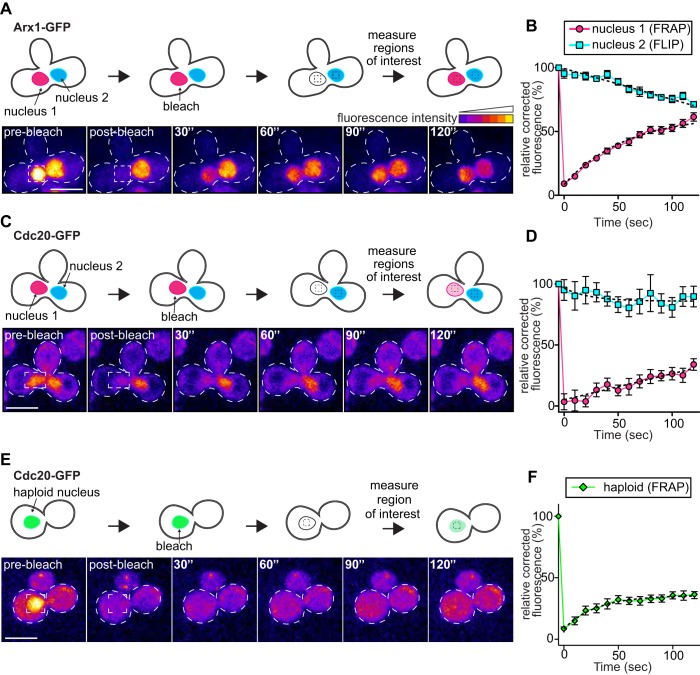
**Cdc20 is restricted to the nucleus during mitosis.** (A,C,E) Schematic of experimental setup along with representative time-lapse images depicting FRAP-FLIP analysis of (A) Arx1-GFP-, (C) Cdc20-GFP-expressing binucleate zygotes or (E) Cdc20-GFP-expressing haploid cells. Fluorescence intensities are displayed as a heat map. (B,D) Relative corrected average fluorescence recovery in the photobleached nucleus (nucleus 1, FRAP; magenta circles), or loss of fluorescence in the unbleached nucleus (nucleus 2, FLIP; blue squares) plotted over time for (B) Arx1- and (D) Cdc20-GFP-expressing zygotes (*n*≥10 binucleate zygotes; see Materials and Methods). (F) Relative corrected average fluorescence recovery in the photobleached nucleus of Cdc20-GFP-expressing haploid cells (green diamonds; *n*≥10 cells). Error bars denote standard error. Curve fits (dashed lines) are one-phase decay non-linear regressions fit to the experimental data. Images were acquired every 10 s for 120 s. Scale bars: 5 μm.

When we photobleached a single Arx1-GFP-containing nucleus in a binucleate cell (Fig. 2A,B; nucleus 1), the GFP fluorescence recovered to 43.0% of its original value after 2 min (after correcting for photobleaching; see Materials and Methods). This is due to the import of unbleached Arx1 into this nucleus. Conversely, the fluorescence intensity of the unbleached Arx1-GFP-containing nucleus (Fig. 2A,B; nucleus 2) decreased by 25.3% after 2 min, indicating that Arx1 molecules from nucleus 2 were actively exported over this time frame. These data are consistent with a previous study, and support the notion that Arx1 is indeed exchanged between nucleoplasm and cytoplasm (Belaya et al., 2006).

In contrast to Arx1, FRAP analysis of Cdc20-GFP revealed a lesser extent of fluorescence recovery in nucleus 1 after 2 min of recovery (27.9%; Fig. 2C,D). More strikingly, FLIP analysis of nucleus 2 revealed almost no loss of fluorescence (4.8%). In light of the minimal fluorescence loss in nucleus 2, we hypothesized that the 27.9% fluorescence recovery of Cdc20-GFP in nucleus 1 was due to the import of Cdc20 molecules from the cytoplasm and not due to export of Cdc20-GFP from nucleus 2. To test this, we performed FLIP-FRAP experiments on Cdc20-GFP-expressing haploid cells, in which nuclear fluorescence recovery can only be due to the import of unbleached proteins from the cytoplasm (and not from Cdc20-GFP molecules from a second nucleus). This analysis revealed that the same degree of fluorescence recovery occurred in these cells as in binucleate zygotes (27.9% after 2 min for both; Fig. 2E,F). Thus, the nuclear fluorescence recovery of Cdc20-GFP in binucleate cells is likely due to the import of protein from the cytoplasm and not to exchange between nuclei. These results suggest that once imported into the nucleus, Cdc20 is not exported back into the cytoplasm. Due to the poor signal-to-noise ratio, we were unable to perform similar experiments with other MCC components (e.g. Mad2 and Mad3). However, given these FLIP-FRAP observations for Cdc20, which is a key component and substrate of the MCC, we postulate that upon nuclear import and subsequent assembly of Cdc20, Mad2, Mad3 and Bub3 into intact MCCs, these complexes remain nuclear confined.

### Multiple spindles within a shared yeast cytoplasm initiate anaphase asynchronously

**Fig. 3.**
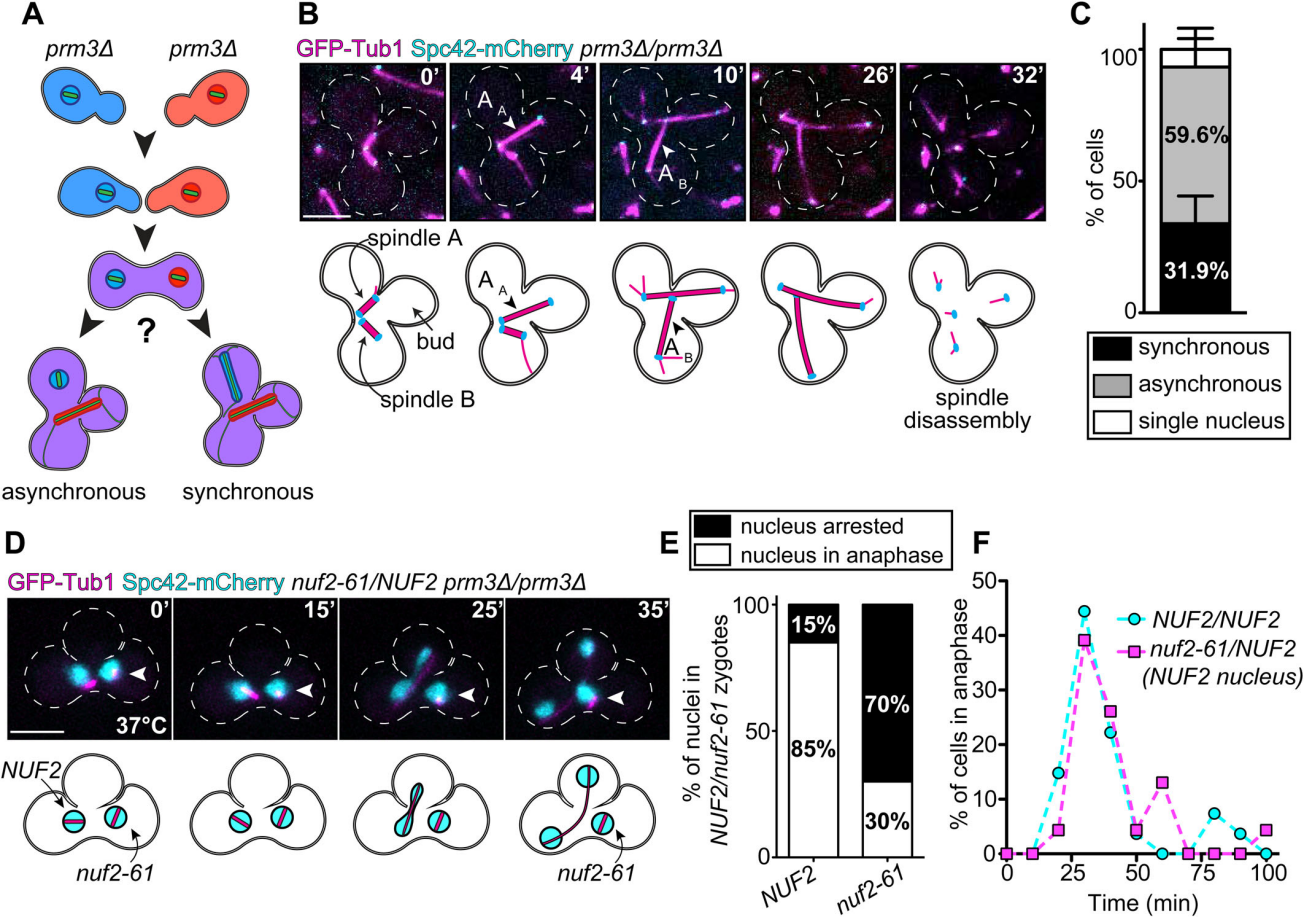
**Anaphase onset occurs asynchronously in binucleate cells.** (A) Schematic of experiment. *MATa prm3*Δ and *MATα prm3*Δ cells were mated together, generating binucleate zygotes. (B) Representative time-lapse images of asynchronous anaphase onset in a binucleate zygotes expressing GFP-Tub1 (magenta) and Spc42-mCherry (cyan). Spindle A initiates anaphase at 4 min (arrowhead, A_A_), while spindle B initiates anaphase at 10 min (arrowhead, A_B_). (C) Plot depicting the frequency with which the indicated anaphase behaviors were observed (*n*=47 binucleate zygotes from four separate experiments). Error bars denote standard deviation. (D) Representative time-lapse images of a *NUF2*/*nuf2-61* binucleate zygote exhibiting asynchronous anaphase onset. White arrowhead denotes the *nuf2-61* expressing nucleus, which was identified as described in the text. Scale bars: 5 μm. (E) Plot depicting the anaphase behavior of *NUF2* and *nuf2-61* nuclei in *NUF2*/*nuf2-61* binucleate zygotes (*n*=27 cells). (F) Plot depicting the timing of mitotic progression as measured from bud emergence (t=0) until anaphase onset (*n*=20 cells for *NUF2/NUF2* zygotes; *n*=15 for *NUF2/nuf2-61* zygotes).

Attenuation of MCC assembly ultimately leads to activation of the APC/C, which in turn triggers anaphase onset (Musacchio, 2015). We reasoned that if MCCs are indeed confined to the nuclear compartment during closed mitosis in yeast, then two mitotic spindles within a binucleate cell would enter anaphase independently of one another (Fig. 3A; asynchronous anaphase onset). In contrast, if MCCs do in fact exchange between nucleoplasm and cytoplasm, then they would also be shared amongst the two nuclei. In such a scenario, anaphase onset would only occur once both spindles achieved proper kinetochore-microtubule attachments and attenuated MCC assembly (Fig. 3A; synchronous anaphase onset).

To distinguish between these two possibilities, we observed mitotic progression in binucleate zygotes expressing GFP-Tub1 (α-tubulin; to delineate mitotic spindles) and Spc42-mCherry (Fig. 3B). Newly formed zygotes were imaged every 2 min as they progressed through mitosis. Anaphase onset was defined as the time at which spindle elongation was initiated (Pearson et al., 2001). Cells were scored as exhibiting synchronous anaphase onset if both spindles initiated anaphase onset within a 2-min imaging window. We found that a significant majority of cells (59.6%) displayed an asynchronous anaphase onset phenotype, in which anaphase onset for each spindle occurred at different times (Fig. 3B,C; *P*=0.0071), whereas a minority (31.9%) exhibited synchronous anaphase onset (also see Fig. S1). In a small number of cells (8.5%), only one nucleus entered anaphase (Fig. 3C; ‘single nucleus’). Fig. 3B depicts representative time-lapse images in which the two spindles within a binucleate cell initiate anaphase at different times (i.e. are asynchronous; spindle A enters anaphase at 4 min, ‘A_A_’, while spindle B enters anaphase at 10 min, ‘A_B_’).

It is worth noting that in a majority of cells (83.3%; *n*=47 zygotes), disassembly of the two mitotic spindles (i.e. mitotic exit) occurred simultaneously (see Fig. 3B and Fig. S1). This process is regulated by the mitotic exit network (MEN), a signaling pathway that ensures that spindle disassembly occurs only when the spindle is properly oriented through the bud neck (Jaspersen et al., 1998; Visintin et al., 1998). Our observations of synchronous mitotic exit are consistent with those from a recent study in which the authors used binucleate zygotes to demonstrate that entry of one SPB into the bud (from one mitotic spindle) is sufficient to activate the MEN, even if both SPBs from the other spindle are situated within the mother cell (Falk et al., 2016; e.g. one spindle is mispositioned; see Fig. S1B). Thus, in contrast to the SAC, for which signals are autonomous for each nucleus, the signal from the MEN generates a dominant signal that triggers mitotic exit in both nuclei.

Although the above data suggest that active MCCs generated in one nucleus cannot diffuse across the nuclear envelope to affect mitotic progression in a neighboring nucleus in a binucleate cell, we sought to explore this further. We employed a strategy in which a SAC-mediated mitotic delay is initiated in only one nucleus within a binucleate zygote, and we then observed how this affected mitotic progression of the other, non-arrested nucleus. To achieve this, we constructed binucleate zygotes containing one nucleus that expressed a functional allele of the NDC80 kinetochore complex component *NUF2*, and one nucleus that expressed a temperature-sensitive *NUF2* allele, *nuf2-61* (Osborne et al., 1994; McCleland et al., 2003). Whereas *NUF2* cells progress through mitosis normally when grown at 37°C, *nuf2-61* cells arrest in mitosis in a checkpoint-dependent manner due to the persistence of incorrect kinetochore-microtubule attachments (see Fig. S2; Osborne et al., 1994; McCleland et al., 2003). We generated binucleate zygotes by mating *NUF2* and *nuf2-61* cells together at 37°C, each of which was expressing a fluorescently labeled allele of the H2B histone (*HTB2*-*TdTomato*, to delineate the nuclei). Only *NUF2* cells possessed the *GFP-TUB1* allele, thereby allowing us to identify the wild-type *NUF2* nucleus prior to zygote formation (not shown); upon cell fusion, however, GFP-Tub1 diffuses throughout the binucleate cell, enabling us to monitor mitotic progression of both nuclei. As expected, the *NUF2* nucleus progressed through mitosis and completed anaphase in most cells imaged (23 out of 27 zygotes; Fig. 3D,E), while the majority of *nuf2-61* nuclei arrested in mitosis (19 out of 27 zygotes; Fig. 3D,E; for the difference between the fraction of arrested nuclei for *NUF2* versus *nuf2-61*, *P*=0.0001). The observation that a fraction of *NUF2* nuclei arrested in mitosis (4 out of 27; Fig. 3E), and a similar fraction of *nuf2-61* nuclei progressed through mitosis (8 out of 27; Fig. 3E) suggested there is some degree of exchange of Nuf2 (or nuf2-61) protein between the two nuclei. We confirmed this is indeed the case by imaging binucleate zygotes in which only one nucleus expressed Nuf2-mCherry (see Fig. S2C,D). We focused our analysis on those cells in which the *NUF2* nucleus progressed through mitotis, and the *nuf2-61* nucleus remained arrested throughout the duration of our experiment (15 out of 27 cells).

We reasoned that if MCCs generated in the *nuf2-61* nucleus could shuttle into the *NUF2* nucleus, then the latter nucleus would exhibit a pronounced mitotic delay relative to wild-type binucleate cells (i.e. those without *nuf2-61*). Thus, we compared the timing of mitotic progression of the *NUF2* nucleus in *NUF2/nuf2-61* zygotes to that of nuclei in *NUF2/NUF2* zygotes by measuring the time from bud emergence until anaphase onset. In *NUF2/NUF2* zygotes, the majority of nuclei entered anaphase within 40 min of bud emergence (mean 34.8 min; *n*=20 cells; Fig. 3F). In *NUF2/nuf2-61* zygotes, the *NUF2* nucleus entered anaphase with similar timing (mean 38.0 min; *n*=15 cells; Fig. 3F). This result demonstrates that the SAC-mediated mitotic delay of the *nuf2-61* nucleus does not affect mitotic progression of the *NUF2* nucleus despite sharing a common cytoplasm. Likewise, because *nuf2-61* nuclei remained arrested in mitosis despite anaphase onset of the *NUF2* nucleus, this suggests that active anaphase-promoting APC/C-Cdc20 complexes are also confined to the nuclear compartment. Taken together, our findings are consistent with a model in which assembled, catalytically active MCCs – and potentially active APC/C-Cdc20 – cannot diffuse from the nucleus in budding yeast.

## MATERIALS AND METHODS

### Strain generation, culture methods and preparation for imaging

All yeast strains were constructed in the BY4743 (Brachmann et al., 1998) background and are listed in Table S1. LRH96 was a gift from Dr Jay Hesselberth (University of Colorado, Anschutz Medical Campus, USA), MMY019 a gift from Dr Michael McMurray (University of Colorado, Anschutz Medical Campus, USA), and strains expressing GFP-SAC effectors were gifts from Dr Santiago DiPietro (Colorado State University, USA). It is worth noting that some of the GFP-tagged alleles used in this study, such as Mad2, are hypomorphic. Because we did not rely on the checkpoint function of these proteins for data interpretation, we did not assess the functionality of these alleles using standard benomyl sensitivity assays. Cells were grown and maintained in rich (YPD) or synthetic defined (SD) media at 30°C (Knop et al., 1999). Transformations were performed using the standard lithium acetate method. Strains expressing fluorescently tagged proteins were gifts (see above) or constructed by either PCR product transformation, plasmid integration or by mating and tetrad dissection. To mate cells for generation of zygotes, parental strains were grown in YPD overnight at 30°C. The next day, approximately equivalent numbers of cells were mixed together in 50 µl of YPD, spotted on a YPD plate, and incubated at 30°C for 3–4 h. Cells were then scraped from YPD plate, washed twice with SD media, and prepared for imaging.

### Plasmid generation

Plasmids used in this study are listed in Table S2. To produce cells with fluorescently-labeled spindle pole bodies, we generated a plasmid that would integrate mCherry::HYG^R^ (encoding hygromycin resistance) at the 3′ end of the *SPC42* locus. To this end, the 3′ end of *SPC42* (nucleotides 699–1089) was PCR amplified using primers flanked with ClaI restriction sites on the 5′ and 3′ ends, digested with ClaI, and ligated into p*mCherry::HYG^R^* digested similarly. This plasmid, pSPC42-mCherry::HYG^R^, was digested with AflII prior to transformation and selection on hygromycin-containing media.

It should be noted that we attempted more direct methods to mark the nuclear envelope in binucleate zygotes for experiments shown in Fig. 1C (i.e. in lieu of Spc42-mCherry). Specifically, we tried to delineate the nuclear envelope or nuclear compartment using mCherry tagged alleles of the nucleoporin Nup133, or the histone Htb2 (the latter of which was used in Fig. 3D). Due to the bright fluorescent signal of both these fusion proteins, and the very dim fluorescence of the GFP-SAC effectors, the mCherry fusions were both apparent at low but detectable levels in the GFP channel, which confounded our localization analysis of the GFP-SAC effector.

### Live cell microscopy

All microscopy was carried out on an inverted Nikon Ti-E microscope equipped with a Perfect Focus unit, a 1.49 NA 100X CFI Plan Apo objective, a piezoelectric stage (for Z-control), an electronically controlled emission filter wheel, an iXon X3 DU888 EM CCD camera (Andor) and a Yokagawa spinning disc head. Excitation light (for imaging and targeted photobleaching) was provided by an AOTF-controlled laser launch with seven lines (Nikon; 405 nm, 445 nm, 488 nm, 514 nm, 561 nm, 594 nm, 640 nm) and two outputs (one dedicated to the spinning disk head, the other to a PA/FRAP unit). The system was controlled by NIS-Elements running on a 64-bit workstation. For time-lapse imaging, cells were perfused into a CellASIC ONIX microfluidics chamber (plate type Y04C, for haploid yeast cells; Millipore). SD media was continuously perfused into the imaging chamber at 7 psi and the chamber was maintained at 30°C throughout the experiment. Step sizes of 0.5 µm were used to acquire 3.5-µm thick Z-stacks every 2, 2.5 or 5 min (as indicated in figure legends). For FRAP-FLIP (see below), cells were spotted onto a 1.7% SD agarose pad. After ∼1 min, a coverslip was mounted on top of the cells, and sealed with paraffin wax.

### Image analysis and processing, and statistical analysis

Time-lapse images were analyzed in both NIS-Elements and ImageJ Fiji (ImageJ, National Institutes of Health) programs. Mitotic spindle lengths were measured and calculated in 3-dimensions. All images presented throughout this study are maximum intensity Z-projections. All brightness and contrast modifications were performed in Adobe Photoshop. Heat-map intensity images presented in Fig. 2 were prepared in ImageJ Fiji after images had been modified in Adobe Photoshop (identical brightness/contrast settings were used for all images within a given experiment). Statistical significance for the data presented in Fig. 3C,E was performed using a Chi-squared analysis in Graphpad Prism.

### FRAP and FLIP

Photobleaching was performed using a 20 mW 405 nm laser at 25% power. After acquisition of a pre-bleach image (exposures: 200 ms, Arx1-GFP; 300 ms, Cdc20-GFP), a single focused 25 ms laser pulse was used to photobleach one nucleus in a binucleate cell. The pulse reduced GFP fluorescence by 70–95%. The extent of fluorescence reduction following the pulse was taken into account when calculating the degree of recovery (43.0% and 27.9% for Arx1- and Cdc20-GFP, respectively). Immediately following the targeted bleach, 0.5 µm step sizes were used to acquire 1.5-µm thick Z-stacks every 10 s for 120 s. Control cells (*n*≥5 cells for both Cdc20-GFP and Arx1-GFP experiments) were subjected to an identical imaging sequence, but without the targeted photobleach pulse. To correct for non-targeted photobleaching, the calculated fluorescence loss in control cells was fitted to a linear regression in Graphpad Prism. The signal loss calculated from the regression equation at each time point was added to both the calculated FRAP and FLIP experimental values. Using ImageJ, the mean fluorescence intensity values for a 5×5 pixel region of interest in each nucleus were corrected for background fluorescence and photobleaching during image acquisition and plotted as the mean intensity with standard error. Graphpad Prism software was used to fit these data to single-decay non-linear regressions.

## Supplementary Material

Supplementary information
