# Supplementary material for: Effectors of the spindle assembly checkpoint are confined within the nucleus of Saccharomyces cerevisiae
Source: Biol Open. 2019 Jun 10;8(6):bio037424. doi: 10.1242/bio.037424 (PMC6602339; doi:10.1242/bio.037424)
Supplement: Supplementary information [file biolopen-8-037424-s1.pdf]

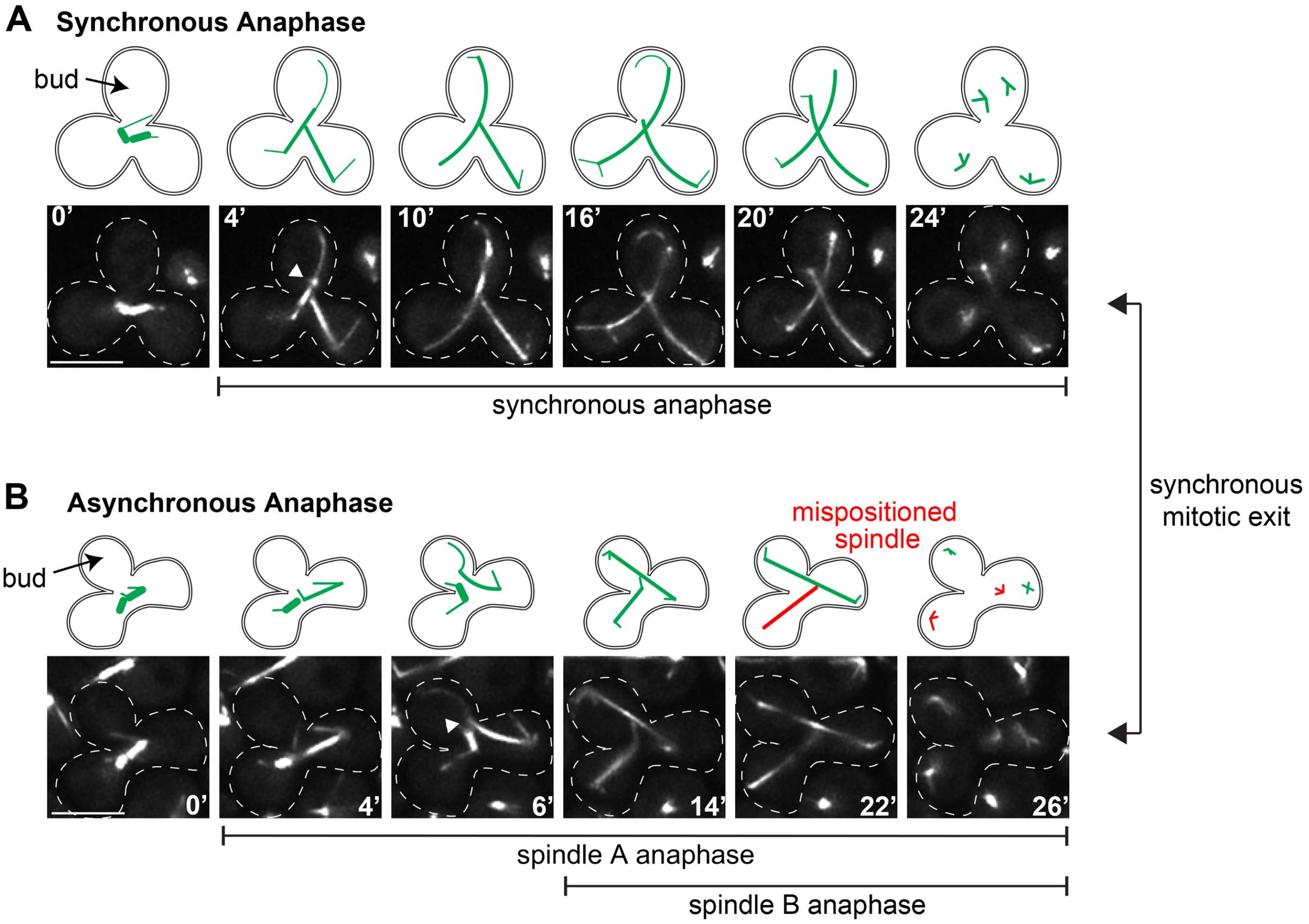

Figure S1. Mitotic exit occurs synchronously in binucleate zygotes. (A) Representative time-lapse images, along with a cartoon model, of mitotic progression in a zygote exhibiting synchronous anaphase onset and mitotic exit. Note that both spindles enter anaphase, and the SPB of one spindle enters the bud at 4' (arrowhead). 20 minutes later, both spindles disassemble, and the cell exits mitosis. (B) Representative time-lapse images, along with a cartoon model, of mitotic progression in a zygote exhibiting asynchronous anaphase onset, but synchronous mitotic exit. In this example, the SPB from the first spindle to enter anaphase enters the bud at 6' (arrowhead), and 20 minutes later, both spindles exit mitosis in spite of one of them (indicated in red) being mispositioned (*i.e.*, it does not extend into the bud).

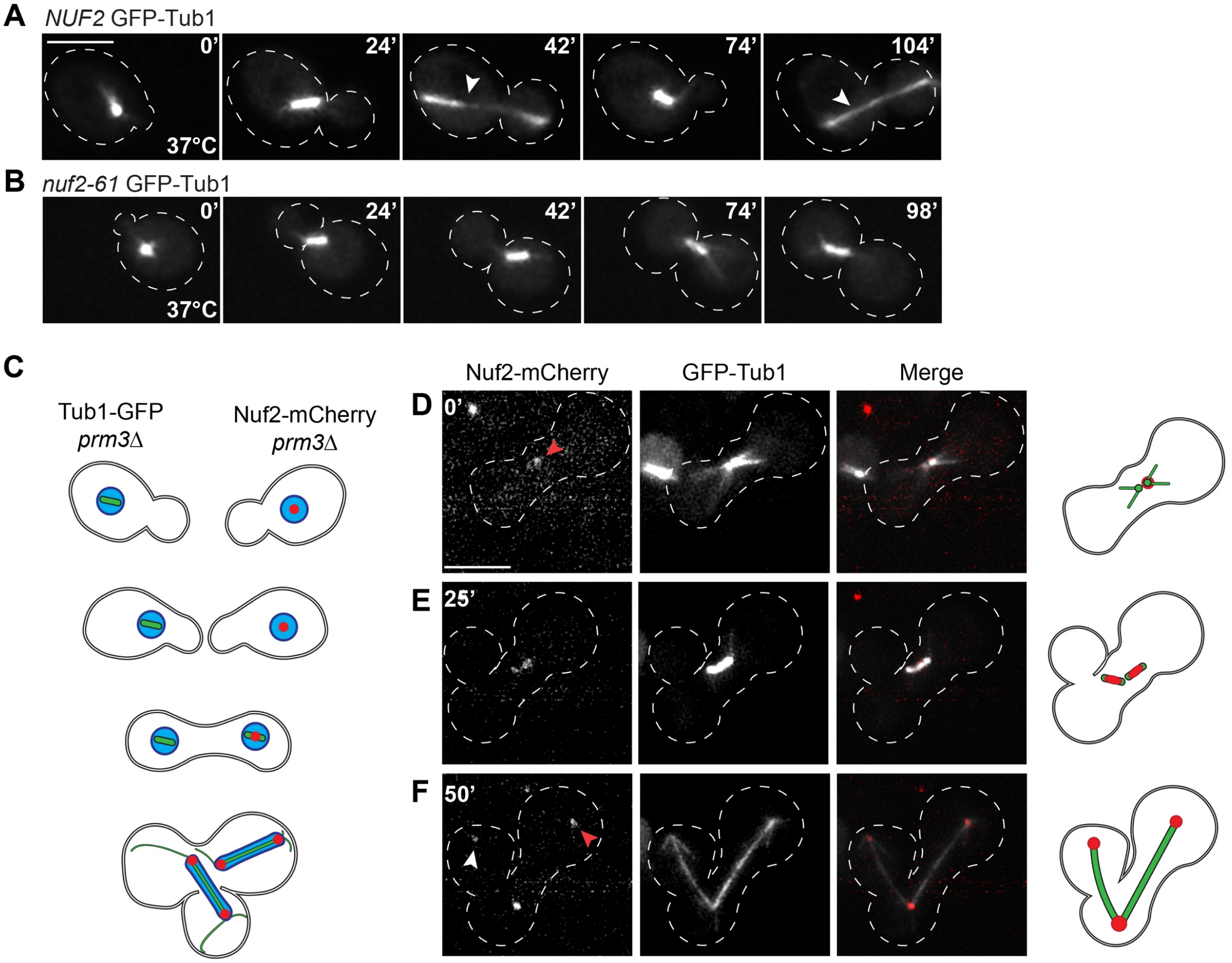

Figure S2. Characterization of nuf2-61 allele and dynamics of Nuf2 protein exchange between nuclei. (A) Representative time-lapse images of a *NUF2* cell progressing through multiple cell cycles at 37°C. Timestamps indicate time from bud emergence. Arrowheads at 42' and 104' indicate anaphase in two consecutive mitoses (separated by ~1 hour). All haploid *NUF2* cells imaged progressed through mitosis at 37°C (n = 50 cells). (B) Representative time-lapse images of a *nuf2-61* cell arresting in mitosis at 37°C. Even after 98 minutes following bud emergence, the pictured cell has failed to enter anaphase, and remains in mitosis with a short mitotic spindle. All haploid *nuf2-61* cells imaged arrested in mitosis at 37°C (n = 57 cells). Cells in (A) and (B) express GFP-Tub1 to visualize mitotic spindles. (C) Schematic of experiment to assess whether Nuf2 protein localizes to both nuclei in a binucleate zygote. (D) Representative time-lapse images (left) and cartoons (right) of a *NUF2/NUF2-mCherry* zygote in which Nuf2-mCherry is detected in both nuclei. At the time of fusion (0'), Nuf2-mCherry is only apparent within the Nuf2-mCherry expressing nucleus (red arrowhead). By 25 minutes following fusion, Nuf2-mCherry is apparent in both nuclei. By 50 minutes following fusion, when both nuclei have entered anaphase, Nuf2-mCherry is apparent at the spindle poles of both mitotic spindles. We noted that the Nuf2-mCherry fluorescence at the spindle pole of the Nuf2-mCherry-expressing nucleus (red arrowhead) is brighter than that of the non-Nuf2-mCherry-expressing nucleus (white arrowhead). Scale bars, 5 µm.

Biology Open • Supplementary information

Table S1: Yeast strains used throughout this study

Strain	Genotype	Source
BY4741	MATa *his3Δ leu2Δ ura3Δ met15Δ*	Brachmann *et al.*, 1998
BY4742	MATα *his3Δ leu2Δ ura3Δ lys2Δ*	Brachmann *et al.*, 1998
LRH25	BY4741 *prm3Δ::HIS3MX6*	This study
LRH26	BY4742 *prm3Δ::HIS3MX6*	This study
LRH29	BY4741 *prm3Δ::HIS3MX6 GFP-TUB1::LEU*	This study
LRH57	BY4742 *prm3Δ::HIS3MX6 SPC42-3mCherry::HPH*	This study
LRH 74	BY4742 *prm3Δ::HIS3MX6 SPC42-3mCherry::HPH TUB1-GFP::LEU*	This study
LRH 75	BY4741 *prm3Δ::HIS3MX6 SPC42-3mCherry::HPH TUB1-GFP::LEU*	This study
LRH 35	BY4741 *MAD1-GFP::HIS3MX6*	Gift from Santiago DiPietro
LRH 58	BY4741 *MAD1-GFP::HIS3MX6 prm3Δ::HIS3MX6 SPC42-3mCherry::HPH*	This study
LRH 81	BY4741 *MAD1-GFP::HIS3MX6 prm3Δ::HIS3MX6*	This study
LRH 36	BY4741 *MAD2-GFP::HIS3MX6*	Gift from Santiago DiPietro
LRH 77	BY4741 *MAD2-GFP::HIS3MX6 prm3Δ::HIS3MX6*	This study
LRH 59	BY4741 *MAD2-GFP::HIS3MX6 prm3Δ::HIS3MX6 SPC42-3mCherry::HPH*	This study
LRH 37	BY4741 *MAD3-GFP::HIS3MX6*	Gift from Santiago DiPietro
LRH 95	BY4741 *MAD3-GFP::HIS3MX6 prm3Δ::HIS3MX6*	This study
LRH 60	BY4741 *MAD3-GFP::HIS3MX6 prm3Δ::HIS3MX6 SPC42-3mCherry::HPH*	This study
LRH 38	BY4741 *BUB1-GFP::HIS3MX6*	Gift from Santiago DiPietro
LRH 75	BY4741 *BUB1-GFP::HIS3MX6 prm3Δ::HIS3MX6*	This study
LRH 96	BY4741 *BUB1-GFP::HIS3MX6 prm3Δ::HIS3MX6 SPC42-3mCherry::HPH*	This study

LRH 40	BY4741 *CDC20-GFP::HIS3MX6*	Gift from Santiago DiPietro
LRH 79	BY4741 *CDC20-GFP::HIS3MX6 prm3Δ::HIS3MX6*	This study
LRH 62	BY4741 *CDC20-GFP::HIS3MX6 prm3Δ::HIS3MX6 SPC42-3mCherry::HPH*	This study
LRH 80	BY4742 *CDC20-GFP::HIS3MX6 prm3Δ::HIS3MX6*	This study
LRH 93	BY4741 *ARX1-GFP::HIS3MX6*	Gift from Santiago DiPietro
LRH 94	BY4742 *ARX1-GFP::HIS3MX6 prm3Δ::HIS3MX6*	This study
MMY0019	BY4741*pdr5 snq2 yor1 HTB2-tdtomato*::HIS3MX	Gift from Michael McMurray
LRH22	BY4742 *prm3Δ::HisMX nuf2-61::kanmx*	This Study
LRH33	BY4742 *prm3Δ::HisMX nuf2-61::kanmx HTB2-TDimer::hisMX GFP-Tub1::LEU2*	This study
LRH34	BY4741*prm3Δ::HisMX HTB2-TDimer::hisMX GFP-Tub1::LEU2*	This study
LRH96	BY4741 *nuf2-61::kanMX*	Gift from Jay Hesselberth
LRH20	BY4742 *prm3Δ NUF2-mCherry::HIS3MX*	This study

Table S2: Plasmids used throughout this study

Plasmid number	Plasmid name	Source
B50	pUC19 GFP-Tub1::*LEU2*	(Maddox et al., 1999)
B217	Spc42-3mCherry::*HPH* (digested with AflII prior to transformation)	This study
